# Unlocking life-threatening COVID-19 through two types of inborn errors of type I IFNs

**DOI:** 10.1172/JCI166283

**Published:** 2023-02-01

**Authors:** Jean-Laurent Casanova, Mark S. Anderson

**Affiliations:** 1 St. Giles Laboratory of Human Genetics of Infectious Diseases, Rockefeller Branch, The Rockefeller University, New York, New York, USA.; 2 Laboratory of Human Genetics of Infectious Diseases, Necker Branch, INSERM U1163, Necker Hospital for Sick Children, Paris, France.; 3 Paris Cité University, Imagine Institute, Paris, France.; 4 Department of Pediatrics, Necker Hospital for Sick Children, Paris, France.; 5 Howard Hughes Medical Institute, New York, New York, USA.; 6 Diabetes Center and; 7 Department of Medicine, UCSF, San Francisco, California, USA.

## Abstract

Since 2003, rare inborn errors of human type I IFN immunity have been discovered, each underlying a few severe viral illnesses. Autoantibodies neutralizing type I IFNs due to rare inborn errors of autoimmune regulator (AIRE)–driven T cell tolerance were discovered in 2006, but not initially linked to any viral disease. These two lines of clinical investigation converged in 2020, with the discovery that inherited and/or autoimmune deficiencies of type I IFN immunity accounted for approximately 15%–20% of cases of critical COVID-19 pneumonia in unvaccinated individuals. Thus, insufficient type I IFN immunity at the onset of SARS-CoV-2 infection may be a general determinant of life-threatening COVID-19. These findings illustrate the unpredictable, but considerable, contribution of the study of rare human genetic diseases to basic biology and public health.

## Introduction

Are there rare and common diseases, or just a myriad of unique expressions of disease in individual patients? The debate between “lumpers,” who favor broad classifications, ranged characteristics, and few divisions, and “splitters,” who favor recognition of nuanced differences, specific characteristics, and many divisions, is long-standing and probably never-ending, but the splitters have gained considerable ground in the last decade, with the impact of the next-generation sequencing of human exomes (1–4). This trend had already become clear by 2010, with the number of known medical conditions expanding from a handful to almost 5,000 in just two centuries. This should come as no surprise to physicians or biologists, as the names we give to diseases are mere labels; the use of words is a fragile attempt to describe a transiently unified perception of a highly heterogeneous and evolving biological reality. Patients are unique, idiosyncratic entities, different not only from each other, but also from themselves at different time points. Even identical twins are not phenotypically identical, and elderly people are different from the youngsters they used to be. The determinism of health and disease operates in living organisms, each of which differs from inert objects in consisting of a unique and diverse collection of cells with somatic genomes evolving both genetically and epigenetically in response to, and with selection due to, the continually changing environment.

Nevertheless, most governments and substantial segments of medical academia insist on categorizing, and even prioritizing medical research, on what they refer to as “common diseases,” as opposed to “rare diseases” (5). Rare diseases are typically defined as conditions affecting fewer than 1 in 2,000 people (in the European Union) or 1 in 1,650 people (in the United States), with common diseases having a frequency above these arbitrary thresholds. Paradoxically, there are many more “rare” than “common” diseases, and it remains unclear whether the total number of patients with “common” disease actually exceeds the number with “rare” diseases. In the industrialized world, this dichotomy both stems from and reinforces a bias toward the study of a few diseases of the elderly, most of which are “common,” at the expense of the many diseases of childhood, most of which are “rare.” COVID-19 constitutes a recent example of a “common disease.” We review here how the enigma of “common” COVID-19, which is essentially a geriatric problem, was cracked at the molecular and cellular levels through the convergence in 2020 of hitherto separate lines of pediatric research on two “rare” genetic conditions: inborn errors of antiviral type I IFN immunity (variants of genes governing type I IFN immunity) and inborn errors underlying the production of autoantibodies against type I IFNs (variants of the *AIRE* gene governing T cell tolerance).

## Inborn errors of type I IFN immunity to viruses

**Figure 1:**
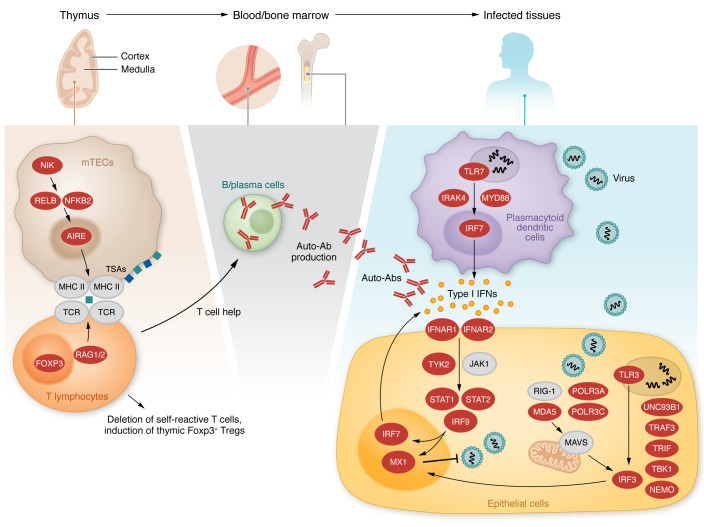
Inborn errors of type I IFN immunity or tolerance. Left, middle: Variants in genes expressed in thymic medullary epithelial cells, indicated in red, are linked to a defect in T cell selection and the production of type I IFN autoantibodies. Right: Variants in genes indicated in red alter type I IFN induction and response pathways.

There are currently 21 human inborn errors of type I IFN immunity (Table 1 and Figure 1).

### Inborn errors of ISGF3 (STAT1, STAT2, and IRF9).

The first human inborn error of type I IFN immunity was reported in 2003, in a child with autosomal recessive (AR) complete STAT1 deficiency presenting with herpes simplex virus encephalitis (HSE) (6). The role of inborn errors of type I IFNs in HSE was not unequivocally demonstrated until almost 20 years later, when a child with HSE due to AR IFN-α/β receptor chain 1 (IFNAR1) deficiency was identified (7). AR complete STAT1 deficiency abolishes both GAS-activating factor–dependent (GAF-dependent) and IFN-stimulated gene factor 3–dependent (ISGF3-dependent) responses to type I, II, and III IFNs, and to IL-27. In total, 24 patients with AR complete STAT1 deficiency have now been reported (8). This condition is the most clinically severe inborn error of type I IFN immunity, with much more serious consequences than AR partial STAT1 deficiency, which has been reported in eight other patients (8). Clinical presentation occurs early in life, and mortality is high. It predisposes patients to a broad range of viral diseases (Table 1). However, it was soon noted that these patients were, paradoxically, not particularly vulnerable to certain common viral infections (9). Only 13 patients with AR complete STAT2 deficiency (10–16) and two with AR complete IFN regulatory factor 9 (IRF9) deficiency (17–19) have been reported. The selective impairment of downstream ISGF3-dependent type I and III IFN responses, with intact GAF-dependent type I, II, and III IFN immunity, probably accounts for the milder clinical phenotype of these patients. They present with a globally and individually narrower range of viral diseases (Table 1).

### Inborn errors of IFNAR1 and IFNAR2.

Evidence that the viral diseases of STAT2- and IRF9-deficient patients result from deficiencies of type I IFN immunity is provided by the similarity of their viral infections to those seen in patients with AR IFNAR1 (7, 20–25) or IFNAR2 (26–29) deficiency. As many as 18 patients with AR IFNAR1 deficiency, and eight with AR IFNAR2 deficiency, have been reported. These patients are rare globally, but about 1 in 1,000 individuals of Western Polynesian or Inuit ancestry are IFNAR1 or IFNAR2 deficient, respectively, owing to the presence of null *IFNAR1* and *IFNAR2* alleles, which are surprisingly “common” (defined as a minor allele frequency greater than 1%) in the Pacific and Arctic regions (22, 29, 30). Surprisingly, only a few viral diseases have been reported in patients with IFNAR1 or IFNAR2 deficiency (Table 1). The most striking wild-type viral illnesses in these patients prior to the COVID-19 pandemic have been HSE and critical influenza. Remarkably, the patients are resistant to most common viruses. The number of patients, their diversity, the small range of viral diseases, their incomplete penetrance, and the occurrence of common deleterious alleles in at least three ancestries all converge to suggest that human type I IFNs are essential for host defense against only a small range of viruses. This observation suggests that there are type I IFN–independent mechanisms of cell-intrinsic antiviral immunity, which may include tissue- and virus-specific restriction factors (31–33).

### Inborn errors of JAK1 and TYK2.

IFNAR1 is constitutively associated with JAK1 and IFNAR2 is constitutively associated with tyrosine kinase 2 (TYK2) (15) in their respective signaling pathways, and patients with AR deficiencies of JAK1 or TYK2 have been reported. AR JAK1 deficiency has been reported only as a partial form in a single patient, who presented a few viral diseases due to its impact on type I IFNs (34, 35). In total, 40 patients with AR TYK2 deficiency have been reported since 2006 (36–41). Two of these patients had a partial defect across pathways, 25 had a complete defect (with or without expression), 3 had a rare selective defect of the IL-23 pathway, and about 1 in 500 individuals of European descent were homozygous for the *TYK2* P1104A variant, which also selectively impairs responses to IL-23. All had severe mycobacterial disease due to impaired responses to IL-23. The cellular response to type III IFN of these AR TYK2-deficient patients appears to be maintained, and responses to type I IFNs are only partially affected, and only in patients with complete or partial TYK2 deficiency, 60% of whom had viral disease (41). Residual type I IFN signaling probably accounts for the relative rarity and benign nature of their viral diseases (Table 1) (16, 41).

### Inborn errors of NEMO, TBK1, IRF3, and IRF7.

Type I IFNs are induced when cells are stimulated or infected, with or without viral replication, and rely on a family of transcription factors and regulators for their production. An AR deficiency in IRF7, a key transcriptional regulator of type I IFNs, was first reported in a 3-year-old child with critical influenza pneumonia (42). AR IRF7 deficiency was recently reported in six other patients from five families (43). Intriguingly, the viral infection phenotype of these patients was restricted to the respiratory tract (Table 1). It is possible that residual IFN-β levels account for the better control of viruses in these patients than in patients with IFNAR1 or IFNAR2 deficiency, despite the lack of IRF7-dependent induction of type I and III IFNs. Adaptive immunity to viruses may also compensate for the defects of type I IFN immunity in these patients (43). In contrast, a child with an autosomal dominant (AD) and partial form of IRF3 deficiency has been reported to have HSE (44). A defect further upstream in the type I IFN–inducing cascade, AD TANK-binding kinase 1 (TBK1) deficiency, also underlies HSE (45). Paradoxically, AR TBK1 deficiency was found to underlie an autoinflammatory condition in four patients aged 7 to 32 years with no history of severe viral disease (46). Finally, a boy with HSE and a specific variant in *NEMO*, encoding the regulatory component of the IKK complex in the canonical NF-κB pathway, has also been reported (47, 48). The mechanism probably involves the disruption of the induction of type I IFN, probably via its impact on IFN-β.

### Inborn errors of TLR3, TRIF, UNC93B, MDA5, and POLR3A/C.

The triggering of type I IFN production frequently relies on viral sensing receptors and their regulators. AR defects of TLR3, TRIF/TICAM1, or UNC93B underlie forebrain HSE (Table 1) (49–52) with a penetrance higher than that of the corresponding AD forms of TLR3 and TRIF deficiency (49, 51–53). TLR3 is an endosomal sensor of double-stranded RNA (dsRNA), which is generated as an intermediate or by-product of viral infection. It also controls the tonic, baseline levels of type I IFN in fibroblasts and cortical neurons, and possibly also in respiratory epithelial cells, with the potential involvement of hitherto unknown endogenous agonists (54). TRIF/TICAM1 is an adaptor and UNC93B is a binding partner in the secretory pathway. TRIF binds almost exclusively to TLR3 (and TLR4), whereas UNC93B is also required for responses to the other three endosomal sensors of nucleic acids, TLR7, TLR8, and TLR9. AR MDA5 deficiency is less rare in the general population, as at least one loss-of-function (LOF) allele has a frequency of almost 1%. However, only four patients with this deficiency have been reported (55–57); three of these unrelated patients presented with respiratory viral diseases other than influenza (55, 57), and the fourth presented with brainstem enterovirus encephalitis (56). TLR3 senses dsRNA in endosomes, whereas MDA5 senses dsRNA in the cytosol. Finally, variants of the genes encoding the dsDNA sensor subunits A and C of RNA polymerase III (POLR3A and POLR3C) have been reported in patients with varicella zoster virus encephalitis (58).

### Inborn errors of TLR7, IRAK4, and MyD88.

Other inborn errors of type I IFN immunity affect the sensing of single-stranded RNA (ssRNA) rather than dsRNA. Almost all patients with AR IRAK4 or MyD88 deficiency described between 2003 and 2019 exhibited pyogenic bacterial infections, but not viral infections (59, 60). Two exceptions were patients with human herpesvirus 6 encephalitis (61, 62). This led to the suggestion that human TLR7, TLR8, and TLR9, which are endosomal sensors of nucleic acids and which all depend on IRAK4 and MyD88 for their signaling, were redundant for host defense against most current and common viruses (59, 60). Moreover, patients with AR UNC93B deficiency, whose cells cannot respond to TLR3, TLR7, TLR8, and TLR9, were found to be prone to HSE, like patients with TLR3 deficiency (49). This further suggested that TLR7, TLR8, and TLR9 were largely redundant in host defense (49). This idea was paradoxical, because the genes encoding the four endosomal TLRs sensing nucleic acids, including TLR3, were under stronger negative selection than those encoding the other TLRs (63). As detailed below, this riddle was solved when X-linked recessive TLR7 deficiency was found to be a genetic etiology of critical COVID-19 pneumonia (64). Patients with IRAK4 or MyD88 deficiency were subsequently found to be at very high risk of life-threatening COVID-19 pneumonia (65). These findings are consistent with the demonstration that plasmacytoid dendritic cells (pDCs) are dependent on IRAK4 and TLR7 for SARS-CoV-2 sensing and type I IFN production (64, 66) and with the observation that patients with chronic lymphocytic leukemia have diminished counts of pDCs and are prone to hypoxemic COVID-19 pneumonia (67).

### Inborn errors of MX1.

The first human inborn error of an IFN-stimulated gene (ISG) to be described was AR ISG15 deficiency (68). The patients did not have viral disease , and their cells were even found to be unusually resistant to viral infection (69). These cells have abnormally high levels of type I IFN activity in vivo, and the patients present a type I interferonopathy manifesting with brain calcifications (69). The underlying mechanism involves unchecked USP18- and STAT2-dependent regulation of the type I IFN response pathway, as confirmed by the identification of patients homozygous for *STAT2* variants disrupting the interaction of STAT2 with USP18 (70–72) and of patients with complete or partial AR USP18 deficiency (73–75), who also have a type I interferonopathy. Paradoxically, the first two recessive disorders of ISGs (ISG15 and USP18 deficiencies) underlie a type I interferonopathy that can potentially increase resistance to viruses. It was not until 2021 that an AD form of MX1 deficiency was reported in Chinese patients with critical disease due to avian influenza virus (76). The IFN-induced GTPase MX1 was first identified by complementation studies in 1986 as essential for immunity to influenza virus in various mouse strains (77). This seminal discovery launched the search for susceptibility genes for host infection (78). Thirty-five years later, an enrichment in rare germline variants of *MX1* was found in Chinese patients with severe avian influenza (76). Most of these LOF variants are also dominant negative.

## Inborn errors of type I IFN tolerance

### APS-1: clinical features and history.

A separate line of research led to the discovery of autoantibodies (auto-Abs) against type I IFNs that impair their activity (Table 2 and Figure 1). Most if not all patients with autoimmune polyglandular syndrome type 1 (APS-1; OMIM #240300), also known as autoimmune polyendocrinopathy ectodermal dystrophy (APECED), develop a defect of type I IFN immunity through an acquired autoimmune response to type I IFNs (79). APS-1 was first described clinically in 1943 (80). It is characterized by the development of multiple organ-specific autoimmune diseases in a single patient, and its inheritance is typically AR. It is globally rare (1 in 200,000), but with a prevalence at least 10 times higher in Scandinavia (1 in 14,000), due to founder effects (81). Autoimmune features vary between individual patients, but the most common clinical features are Addison’s disease, hypoparathyroidism, and an unusually selective susceptibility to chronic mucocutaneous candidiasis (CMC). This core triad is seen in about 75% of patients. Even within families, the autoimmune conditions that develop may differ between affected relatives. The management of APS-1 patients typically involves supportive care and, frequently, replacement therapy for affected organs, with immunosuppression occasionally used to treat more severe features, such as autoimmune hepatitis (82). The overall clinical outcome of APS-1 patients is highly variable, but mortality reaches 50% by the age of 45 years, typically due to the cumulative effect of multiple disease features and their sequelae (83).

### The discovery linking AIRE to APS-1.

Given the typical AR pattern of inheritance for APS-1, physical linkage approaches mapped the defective gene to human chromosome 21 in 1994 (84). Continuing with this laborious linkage approach, two groups simultaneously reported the identification of the defective gene in 1997. It was agreed to name the gene “autoimmune regulator” (*AIRE*), given the clinical phenotype of APS-1 patients (85, 86). This new gene displayed no marked sequence similarity to any known gene and was thought to encode a 545–amino acid protein with at least four distinct domains. Analysis of the sequence of the *AIRE* gene indicated that it contained a nuclear localization domain (85, 86). In addition, staining for the protein resulted in a speckled nuclear pattern in cells actively expressing the gene (87). This critical gene hunt paved the way for the unlocking of a critical regulator of immune tolerance, because patients with this disease harbored variants predicted to be loss-of-function (e.g., nonsense variants) when homozygous. It was not until 2014 that heterozygous and dominant-negative variants of *AIRE* were found to underlie an AD form of APS-1, in both multiplex and sporadic families (88–90), typically with a milder phenotype.

### Immunological role of the AIRE gene product.

Major clues to the function of AIRE were initially provided by studies mapping its expression to the thymus and, particularly, to the medullary thymic epithelial cells (mTECs) (91). A knockout mouse model was developed that also presented multiple autoimmune conditions (91). A detailed analysis of mTECs in the knockout mouse gave rise to a model in which AIRE promotes the expression of a wide array (i.e., thousands) of tissue-specific self-antigens (TSAs), all expressed in isolated tissues (91, 92). Another interesting picture of gene expression is being unraveled in mTECs, a fraction of which further differentiate after AIRE expression and acquire a gene expression program reflective of some peripheral tissues, including enteroendocrine, respiratory epithelium, mature skin epithelium, and tuft cells, that also contribute to TSA expression (93–96). During development, thymocytes traffic through the medullary compartment, where these cells undergo a critical negative selection step in which self-reactive T cells are eliminated by the recognition of self-antigens in the medulla. AIRE controls T cell immune tolerance by driving the expression of “self” within the medulla such that self-reactive T cells that develop by chance can recognize self and be eliminated from the developing T cell pool (Figure 1). AIRE can also promote the development of a fraction of CD4^+^ Foxp3^+^ Tregs protective against autoimmunity in the periphery (97). Studies in mice have clearly established that this elegant thymic selection process is remarkably efficient and frequently results in the deletion of tissue-specific T cells (98). Interestingly, recent single-cell studies of the human thymus have also demonstrated that TSAs are expressed in AIRE-expressing cells within the thymus and that these cells frequently express the targets of the autoimmune response in APS-1 patients (94).

### Mechanisms of autoimmune endocrine and fungal diseases.

The core mechanism of disease in APS-1 patients is T cell driven, but tissue damage is often associated with tissue-specific auto-Abs. Endocrine disease in APS-1 patients is mostly driven by a T cell–mediated destruction of the affected organ, and mouse models have provided support for a prominent Th1-like response in affected tissues (99). APS-1 patients can develop a wide array of autoimmune responses, but, unexpectedly, some of these responses were found to be directed against cytokines. As mentioned above, CMC is an intriguing prominent condition in APS-1 patients. CMC develops in these patients due to an autoimmune response directed against crucial Th17 cytokines, such as IL17A and IL17F, with auto-Abs neutralizing both these cytokines detected in most patients (100, 101). This autoimmune connection is further bolstered by data showing that candidiasis frequently develops in patients with germline variants affecting IL-17A/F and its IL17RA/RC receptor (102) and in patients treated with blocking antibodies against these cytokines for inflammatory conditions (103). These findings suggest that most if not all of the clinical features of APS-1 patients, including their characteristic isolated fungal infection, are of an autoimmune nature.

### “Silent” auto-Abs against type I IFNs.

It was reported from 2006 onward that more than 90% of patients with APS-1 develop auto-Abs against type I IFNs (104). They have also been found in patients with myasthenia gravis, thymoma, and systemic lupus erythematosus, and in individuals treated with IFN-α2 or IFN-β (105–108). The clinical significance of these auto-Abs generally, and in APS-1 patients in particular, remained unknown, as patients with these auto-Abs displayed no consistent susceptibility to viral infections. The auto-Abs against type I IFNs observed in individuals with APS-1 are almost exclusively directed against the 13 IFN-α forms and the single ω form, rarely against IFN-β, and apparently not against ε and κ (104). This pattern was identified as a possible reason for the lack of overt association of these antibodies with a viral susceptibility phenotype. IFN-β, in particular, is the first type I IFN induced by viruses in most cells. As detailed below, it was not until 2020 that APS-1 patients were found to be at very high risk of critical COVID-19 pneumonia and even other viral diseases (109, 110). The high prevalence of auto-Abs against type I IFNs in patients with APS-1 and thymoma suggests that defects of thymus function may trigger this specific autoimmune response. In support of this notion, *AIRE* expression has been shown to be impaired in thymoma, connecting the mechanism of autoimmunity in patients with inherited APS-1 with acquired thymoma (111).

### Other mTEC etiologies of auto-Abs against type I IFNs.

AIRE expression in mouse mTECs is driven by RANK via the alternative NF-κB pathway (Figure 1) (111–114). Consistently, auto-Abs against type I IFNs were found in patients with AR NIK or RELB deficiencies, and patients with a specific form of AD NF-κB2 deficiency due to C-terminal variants preventing the cleavage of p100 into p52, resulting in a loss of p52 activity but a gain of inhibitory function for p100 (115). Moreover, *AIRE* expression in the thymus was found to be impaired in the patients with *RELB* or *NFKB2* variants studied. Deficiencies of the alternative NF-κB pathway can, therefore, underlie the production of auto-Abs against type I IFNs through an impairment of AIRE expression in mTECs. By contrast, the patients with inborn errors of canonical NF-κB immunity tested had no auto-Abs against type I IFNs. However, most women with incontinentia pigmenti due to heterozygosity for LOF *NEMO* variants do have such auto-Abs, possibly due to the apoptosis of mTECs expressing the mutated *NEMO* allele during thymic development (116). Collectively, these findings suggest that AIRE-dependent thymic dysfunctions (deleterious variants of *AIRE* or the genes encoding components of the AIRE-inducing pathway in mTECs, or locally, within a thymoma) may underlie the production of auto-Abs against type I IFNs.

### Other T cell etiologies of auto-Abs against type I IFNs.

Several T cell–intrinsic inborn errors have also been found to underlie auto-Abs against type I IFNs. Male patients with deleterious variants of the X-linked gene *FOXP3*, who display a loss of functional Tregs, often carry auto-Abs against type I IFNs (117). They present with a condition known as immune dysregulation, polyendocrinopathy, enteropathy, X-linked (IPEX) (118, 119), which has autoimmune and clinical features partly overlapping with those of APS-1 (120). They have not been reported to have severe viral disease, at least before immunosuppressive therapy for hematopoietic stem cell transplantation. Patients with *RAG1* or *RAG2* deleterious variants and combined immunodeficiency may also produce auto-Abs against type I IFNs (121). These patients frequently have herpes virus diseases, due to the presence of these auto-Abs against type I IFNs alone or together with the combined T and B cell deficiency. The known etiologies of auto-Abs against type I IFN thus affect T cell tolerance, in a T cell–intrinsic manner (RAG, FOXP3) or via mTECs (AIRE and the pathway that induces it). AIRE defects are linked to impairment of the correct selection of Tregs (97), and defects of RAG1 and RAG2 are linked to impairment of the expression of AIRE (122). Together, these data again link the generation of auto-Abs against type I IFNs to thymic selection.

## Critical COVID-19 pneumonia and type I IFN deficiency

### The problem, hypothesis, and approach.

The key problem posed by COVID-19 in 2020 is common to all human pathogens: what drives the vast interindividual clinical variability observed during infection (78, 123)? The global infection fatality rate (IFR) of COVID-19 in unvaccinated individuals was about 1% across all ages and sexes. The risk of death was found to double every 5 years of age, from childhood onward, accounting for the risk of death being 10,000 times greater at age 85 than at age 5 (124). We hypothesized that critical COVID-19 pneumonia might result from single-gene inborn errors of immunity, at least in some patients (125). The identification of a causal inborn error, even in a single patient, might be sufficient to pull the mechanistic thread to reveal other causes disrupting the same physiological mechanisms in other patients (78). The COVID Human Genetic Effort (www.covidhge.com) was set up to follow this approach and to enroll as many patients as possible worldwide, such that even low levels of genetic homogeneity could be detected (125). The phenotypes and genotypes of the patients were made available to all the teams of the consortium, facilitating coordinated and synergistic research into the human genetic and immunological determinants of critical COVID-19.

### Inborn errors of immunity to influenza and candidate genes.

The first hypothesis tested was that critical pneumonia due to seasonal influenza virus and critical pneumonia due to SARS-CoV-2 might be allelic. Patients with AR IRF7 deficiency, AR IRF9 deficiency, and AR or AD TLR3 deficiency were prone to severe influenza (126). Another ten genes were considered, with (a) products biochemically and immunologically connected to the three core influenza susceptibility genes and (b) germline variants already shown to underlie other severe viral illnesses (Figure 1). The genes considered included those encoding STAT1 and STAT2, which were soon confirmed to be influenza susceptibility genes (8, 14). Rare and deleterious variants of 8 of the 13 candidate genes were found in 23 patients with critical COVID-19 pneumonia. Eleven patients had known dominant disorders, whereas eight had potentially new dominant disorders. These findings were replicated in a larger cohort (127). Germline variants affecting the TLR3 pathway suggested that tonic type I IFN levels in respiratory epithelial cells (RECs) played an important role in host defense against SARS-CoV-2 (54). Four patients with AR defects provided unique insight into the pathogenesis of COVID-19. Two unrelated adults were found to have AR IFNAR1 deficiency, while another two had AR IRF7 deficiency (23). Other patients with critical COVID-19 due to AR IFNAR1 (16, 24, 25) or AR IRF7 (43) deficiency were later reported, as well as a patient with AR TBK1 deficiency (128). Remarkably, the young and even middle-aged adults with such profound AR deficits identified had remained well until they developed COVID-19.

### Genome-wide search: TLR7 and type I IFN again.

A burden test on the X chromosome found an enrichment in rare non-synonymous variants at a single locus encoding the endosomal RNA sensor TLR7 (64). The lack of enrichment at the X-linked *TLR8* locus suggested not only that most TLR7 variants were deleterious and pathogenic, but also that the mechanism of disease involved a disruption of the TLR7-dependent induction of type I IFN by pDCs. Indeed, TLR7 and TLR8 are both endosomal sensors of overlapping RNAs, and both signal via the MyD88- and IRAK4-dependent signaling pathway, which had already been shown to be essential for SARS-CoV-2 sensing in pDCs; however, TLR7 is expressed in pDCs, whereas TLR8 is not (66). Further experiments showed that most TLR7 variants in patients with critical COVID-19, but none of those in mildly infected individuals, were LOF. Penetrance was incomplete among relatives of index cases. TLR7-deficient pDCs had profoundly impaired responses to SARS-CoV-2 (64). X-linked recessive TLR7 deficiency was found in about 1% to 2% of male patients with critical COVID-19. The proportion of adults with critical pneumonia due to these 14 inborn errors, including autosomal defects, was about 3% to 5%, while about 10% of children with COVID-19 pneumonia had recessive deficiencies not only of TLR7 and IRF7, but also of STAT2 and TYK2 (16). An unbiased, genome-wide approach yet again implicated defects of type I IFN immunity. TLR3 pathway variants had implicated resident RECs, but TLR7 variants implicated circulating pDCs, implying that the recruitment of these cells to the respiratory tract during SARS-CoV-2 infection was essential for type I IFN–mediated protective immunity.

### APS-1 patients and hypoxemic COVID-19 pneumonia.

Early in the COVID-19 pandemic, several patients with APS-1 developed critical COVID-19 pneumonia (116, 129). Given this knowledge and the identification of inborn errors of type I IFNs in other patients with critical COVID-19, a unifying hypothesis developed according to which the susceptibility to critical COVID-19 pneumonia of APS-1 patients was due to their preexisting auto-Abs against type I IFN. In an international series of 22 APS-1 patients aged 8 to 48 years, 86% had hypoxemic pneumonia, including 68% with severe disease and 18% who died (109). A smaller, single-center study of four patients confirmed that not all patients with APS-1 infected with SARS-CoV-2 developed hypoxemic pneumonia (130), while a more recent study reported several other APS-1 patients with critical COVID-19 (131). Importantly, the auto-Abs against type I IFNs were present in the APS-1 patients before their infection with SARS-CoV-2 and the development of COVID-19 pneumonia. Given that inborn errors of type I IFN immunity have been shown to be causal for critical pneumonia, these findings provided proof of principle that auto-Abs neutralizing type I IFN may also be causal for critical pneumonia. This rare disease therefore provided a key insight into one of the possible mechanisms underlying the development of severe course of COVID-19 in some subjects.

### Auto-Abs against type I IFNs in patients with critical COVID-19.

Remarkably, about 10% of patients with critical COVID-19 carried circulating auto-Abs neutralizing high concentrations of IFN-α and/or IFN-ω (116). This proportion was subsequently found to be higher (15%) if patients whose plasma neutralized lower concentrations were considered (132). Auto-Abs neutralizing IFN-β were rarely found. Patients with auto-Abs against type I IFNs collectively accounted for 20% of deaths across age groups and 20% of critical cases among patients over 70 years of age. The risk of critical disease increased with both the number of forms of type I IFN and the concentration of IFN neutralized (132, 133). These findings have been replicated in 29 independent populations worldwide (109, 130, 131, 134–159). These auto-Abs have also been shown to underlie a delayed type I IFN ISG response in leukocytes, as shown by single-cell RNA sequencing (156), and in the nasal mucosae, as shown by RNA-Seq (160). These auto-Abs were detected in blood samples drawn very early during hospitalization (156) and even in pre–COVID-19 samples for the small number of patients for whom such samples were available. Their levels in the blood may increase during COVID-19 (136, 161). Their prevalence was studied in 33,000 individuals aged 20 to 100 years for whom samples collected before 2019 were available (132). Their prevalence remained stable until the age of 65 years, between 0.3% and 1% depending on the concentrations neutralized, subsequently increasing to reach 4% and 7%, respectively, after the age of 80 years (132). The prevalence of auto-Abs neutralizing IFN-β remained stable, at about 0.2%, across age groups. These findings suggest that the IFR is much greater in individuals with auto-Abs than in those without these antibodies (133).

### Developing a clinical test: risk stratification and treatment approaches.

The presence of these auto-Abs is the second most important common risk factor for critical COVID-19 after age. If the risk associated with age and the risk associated with the presence of auto-Abs against type I IFNs are combined, the effective mortality rate for COVID-19 can reach levels well over 50% in subjects over 80 years old carrying auto-Abs against type I IFNs (133). This provides a strong argument for testing for these antibodies in the initial assessment of patients diagnosed with COVID-19, especially, but not exclusively, in those who have not been vaccinated. We recently found that about 20% of cases of “breakthrough” hypoxemic pneumonia were due to auto-Abs neutralizing high concentrations of both IFN-α and -ω, despite good antibody responses to the RNA vaccine and a normal capacity to neutralize the virus (162, 163). The development of a simple screening test in the clinical setting for widespread deployment with a quick turnaround time is warranted. A positive result on such a test in healthy individuals would have implications for vaccination (influenza, COVID-19) and follow-up, and would contraindicate certain other vaccinations (e.g., the yellow fever vaccine YFV-17D). It would also have implications for rapid correct treatment in patients diagnosed with specific viral infections. For example, it will be interesting to see whether IFN-β treatment is a feasible approach (116). Recent trials with IFN-β revealed little evidence of the benefit of such treatment in hospitalized patients (164), but trials in an ambulatory setting are warranted.

### A general mechanism of viral disease.

Auto-Abs neutralizing type I IFN were recently shown to underlie severe herpes simplex or zoster virus disease in patients hospitalized for COVID-19 (137). These findings are consistent with the seminal report by Ion Gresser and colleagues of auto-Abs against type I IFN in a 77-year-old woman with disseminated shingles (165) and with the occurrence of such viral infections in patients bearing deleterious genotypes of *RAG1* or *RAG2* or carrying such auto-Abs (121). A similar observation was recently made in a large cohort of patients with systemic lupus erythematosus (SLE) (148). Moreover, one-third of the small series of patients with adverse reactions to the live attenuated YFV-17D vaccine had such auto-Abs (28). Remarkably, these patients included a young woman subsequently diagnosed with SLE, an elderly woman, and an elderly man. These three groups are at risk of producing auto-Abs against type I IFNs and had already been shown to be at greater risk of adverse reactions to YFV (166). Finally, about 5% of patients under 70 years of age carried such auto-Abs, and the estimated risk of critical influenza increased with the concentration and number of IFNs neutralized (167). Other candidate viral diseases for which auto-Abs against type I IFNs increase susceptibility include the viral infections seen in patients with inborn errors of type I IFN immunity. The evidence of a role of auto-Abs against type I IFNs is already clear for at least four viral illnesses: critical COVID-19 pneumonia, influenza pneumonia, adverse reactions to the YFV-17D vaccine, and recurrent or disseminated shingles.

## Concluding remarks

The discovery of inborn errors of type I IFNs and auto-Abs against these cytokines in at least 15% to 20% of patients with critical COVID-19 pneumonia suggested a unifying general mechanism of disease (78, 124). The “common” COVID-19 enigma was cracked thanks to previous studies conducted over several decades on two groups of patients with “rare” and seemingly opposite Mendelian phenotypes: infectious and autoimmune (78). This rare-to-common, patient-to-population, genetic-to-mechanism approach (78, 124, 168) contrasts with other approaches. The population-based approach to COVID-19, in which this “common disease” is tackled via purely mathematical (genetic association studies) or purely immunological (blood or mucosal multi-omics) approaches, met with less success. Rather than detecting the immunological causes of viral disease, the latter studies analyzed immune responses to the virus (124). Rather than detecting the genetic causes of viral disease in individual patients, the former studies detected common modifiers of disease at the population level. We argue that, with the splitters’ approach, focusing on individual patients and “rare diseases,” particularly in young patients, with individual human beings seen as single organisms, it can be possible subsequently to lump patients with different causes of disease via shared mechanisms together. By contrast, with the lumpers’ approach, focusing on large populations and “common diseases,” mostly in elderly populations, it is not easy to split patients into different groups later, owing to the lack of unambiguously identified causes and mechanisms of disease. Studies of “rare” outliers constitute a powerful approach that can be used to guide the exploration of “common” diseases, whether viral or otherwise (78, 168–171).

## Figures and Tables

**Table 2:**
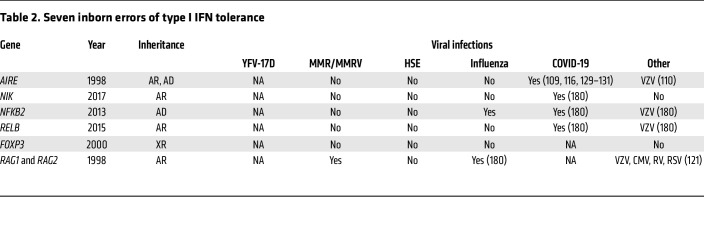
Seven inborn errors of type I IFN tolerance

**Table 1:**
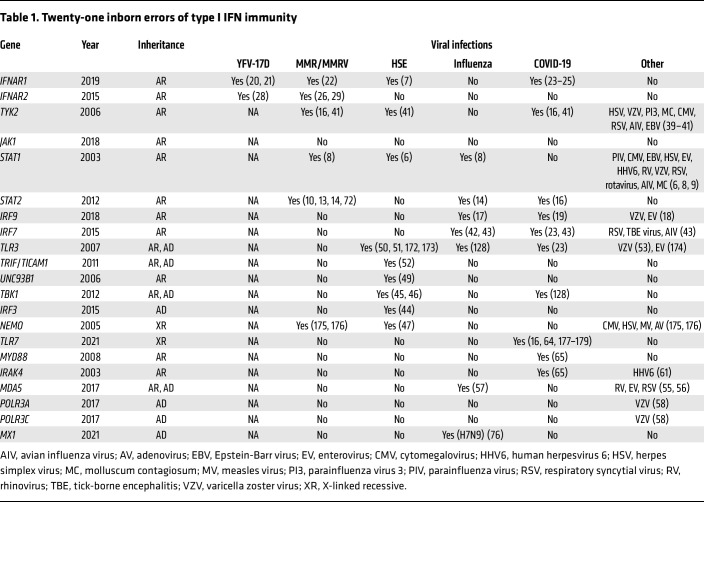
Twenty-one inborn errors of type I IFN immunity

## Version 1 02/01/2023

Electronic publication
